# Creating a Workplace Culture of Preventive Health: Process and Outcomes of the Colon Cancer–Free Zone at Virginia Cooperative Extension

**DOI:** 10.1007/s13187-019-01569-4

**Published:** 2019-07-15

**Authors:** Carlin L. Rafie, Lindsay Hauser, John Michos, Jeffrey Pinsky

**Affiliations:** 1 Department of Human Nutrition, Foods and Exercise, Virginia Polytechnic Institute and State University, 321 Wallace Hall (0430), 295 West Campus Drive, Blacksburg, VA 24061 USA; 2 University of Virginia Cancer Center, 1215 Lee Street, Charlottesville, VA 22903 USA; 3 Anthem Blue Cross and Blue Shield of Virginia, 2015 Staples Mill Rd, Richmond, VA 23230 USA

**Keywords:** Colorectal cancer, Worksite wellness, Cancer screening, Cooperative extension, Cancer prevention

## Abstract

Colorectal cancer is the third leading cause of cancer death in the USA, yet is highly preventable and detectable at an early stage through screening. Virginia Cooperative Extension (VCE) implemented a worksite colon cancer awareness program to increase colorectal cancer screening rates and preventive lifestyle behaviors among its employees. The Colon Cancer–Free Zone program is designed using best practice principles of worksite health programs and includes information sessions covering the topics of colorectal cancer, screening guidelines, insurance coverage, and preventive lifestyle behaviors. It is conducted in a campaign format that includes a strategic communication strategy targeting relevant screening barriers and facilitators, peer champions, and incentives. The program was implemented with VCE employees statewide utilizing a web-based system for the information sessions, and resulted in broad participation, a significant increase in screening self-efficacy (4.15 ± 0.64 vs 3.81 ± 0.76, *ρ* = 0.006), changes in diet and physical activity (50% and 40% of participants, respectively), and a 20.6% increase in the employee colorectal cancer screening rate. A Colon Cancer–Free Zone toolkit was developed for use by Extension Agents to implement the program at worksites in their service communities.

**Electronic supplementary material:**

The online version of this article (10.1007/s13187-019-01569-4) contains supplementary material, which is available to authorized users.

## Background

Colorectal cancer (CRC) is the third leading cause of cancer-related death in the USA among men and women; yet it is highly preventable and detectable through healthy lifestyles and screening [1, 2]. New screening methods have allowed for the early detection of colorectal cancer, and its prevention through the removal of precancerous intestinal polyps. Early detection of colorectal cancer is crucial to successful treatment and increases survival rates from 13.1% when discovered at an advanced stage to 90% if found at an early, localized stage [3]. In addition to screening, a healthy lifestyle that includes regular exercise, a healthy eating pattern, and appropriate weight maintenance is pivotal to a decreasing risk for colorectal cancer [2]. Innovative systems to bring sustained colorectal cancer screening and prevention information, particularly to at-risk populations, could make a significant impact on the incidence and outcomes of colorectal cancer in the USA.

Interventions aimed at increasing colorectal cancer screening have targeted providers, health care systems, and direct patient education in community-based settings [4]. Appropriately adapted community education programs are needed to address knowledge gaps, negative attitudes, and other barriers to screening and healthy lifestyles in the population and are an important part of a comprehensive strategy for reducing colorectal cancer incidence and increasing screening rates. Such programs exist, but require further theory-based evaluation of effectiveness and implementation within existing systems of public education, including the Cooperative Extension Service, departments of health, and corporate and healthcare wellness programs, among others [4].

The Cooperative Extension System (CES) is a nationwide education and outreach network established in 1914 that uses research findings emanating from the over 100 state land-grant colleges and universities to equip Americans in rural and urban communities with the knowledge and skills to solve community, farm, and family problems. The CES has been providing evidence-based education in almost every county in the USA for over 100 years [5]. Recently, health and wellness has become a programmatic focus. Cooperative Extension’s National Framework for Health and Wellness was published in 2014 in response to national trends in health and in recognition that the Extension has the community presence and local credibility needed to influence the social, economic, and environmental determinants of health [6]. The Framework is aligned with the US Department of Health and Human Services’ National Prevention Strategy and recognizes the need for CES to leverage existing partnerships and establish new public and private partners from health, education, and preventive service areas (see Supplementary Materials) [4, 7].

In line with the Framework, Virginia Cooperative Extension (VCE) joined a national effort to eliminate colorectal cancer as a public health issue, the National Colorectal Cancer Roundtable (NCCRT) 80% by 2018 initiative, in February 2016 [8]. There is a growing body of evidence for the efficacy of group education conducted within worksites to promote colon cancer prevention and screening [9–13]. We implemented a program to increase CRC screening rates of VCE employees and create capacity within family and consumer science extension agents to conduct colorectal cancer screening and risk reduction programs at worksites in their service communities. This paper reports on the process and outcomes of the program conducted within VCE.

## Methods

We implemented a two-phase program to increase VCE screening rates and bring CRC prevention awareness to Virginia communities. The Colon Cancer–Free Zone (CCFZ) is designed using best practice principles of worksite health programs including acquiring administration buy-in, developing a strong communication strategy appropriate to the corporate culture, use of peer champions, and provision of relevant incentives for employee participation [14, 15]. It has four key elements (1) a series of colon cancer risk reduction and screening information seminars, (2) a communication plan targeting screening barriers, (3) peer colon cancer champions, and (4) a call to personal action with incentives. We implemented the program within VCE to employees statewide and subsequently developed a CCFZ toolkit for Extension Agents to conduct the program within worksites in their service counties.

### VCE CCFZ Program Implementation

Prior to implementation of the program, top administrative personnel announced that VCE had joined the National 80% by 2018 initiative during the annual VCE Spring conference and encouraged all employees to engage in the CCFZ activities being planned to increase CRC awareness and screening. We distributed a survey previously developed to identify factors associated with CRC use and based on health belief model (HBM) constructs [16] to VCE personnel during the annual conference. Results from the survey were used to target information provided during the organization-wide program and to form messages for the communications plan developed for the program. Four interactive, web-based information sessions were conducted across 4 months starting in May 2016 and addressed (1) CRC screening guidelines and insurance coverage, (2) dietary habits and weight management to reduce CRC risk, (3) physical activity guidelines and recommendations for CRC risk reduction, and (4) program information review and personnel testimonials. Sessions were facilitated by an Extension Specialist and topic experts.

To encourage VCE employee participation across the state, “Colorectal Cancer Champions” were recruited from employees in each of the 4 state VCE districts. These individuals volunteered as champions and had an interest in the topic due to experience, either personal or among family or friends, with colorectal cancer. They transmitted information about upcoming information sessions, distributed social media messages that were part of the program, and encouraged conversation about colorectal cancer within their district offices. The communication plan was developed using guidance provided by a communications guidebook developed by the NCCRT [17], information from the pre-program survey, and key messages being promoted by the National campaign.

A program website, Facebook page, and Twitter account were created to facilitate communication. The national key message that colorectal cancer is preventable, treatable, and beatable was repeated in all communication platforms. Facebook posts and tweets were scheduled to be delivered across 6 months, starting 2 weeks prior to the first information session. Motivational messages appropriate for our target audience of unscreened individuals with health insurance were provided through these platforms and included messages addressing control, expectation, trust, and empowerment. Testimonials of colleagues were also posted on the webpage and Facebook page. In addition, a poster announcing the VCE 80% by 2018 program was produced and provided to all 107 county Extension offices for posting.

Finally, employees were encouraged to sign a CCFZ “pledge” as a call to action. The pledge was available in both paper format distributed by the Colorectal Cancer Champions and electronically on the website. Signers pledged to get screened for colorectal cancer if they were eligible, to know three simple facts about colorectal cancer provided on the pledge card and to talk to three additional people about colorectal cancer prevention and screening.

## Data Analysis

### Pre-/Post-VCE CCFZ Program Survey

A validated twenty-eight question health beliefs survey evaluating factors associated with CRC use and demographics of respondents was distributed to the VCE personnel in February 2016 during the in-person annual VCE conference [16]. The survey was repeated in February 2017, with three additional questions about awareness of the CCFZ campaign, participation in CCFZ activities, and actions taken to get screened. The VCE annual conference was conducted virtually in 2017, so distribution of the post-survey was conducted through e-mail solicitation of VCE employees.

The pre- and post-survey evaluated the attitudes of respondents around five health belief categories; self-efficacy (4 questions), benefits of screening (5 questions), barriers to screening (6 questions), susceptibility to colorectal cancer (3 questions), and severity of colorectal cancer (4 questions). Cronbach’s alpha analysis indicated internal consistency among questions within each belief category (all α ≥ 0.710), so composite scores were calculated for each belief category. To accomplish this, responses to each question were given a number from 1 to 5, with “strongly disagree” as 1 and “strongly agree” as 5. In the case of questions where agreement indicated a health belief associated with negative engagement in colorectal cancer health behaviors, the number ordering was reversed. The average score for questions within each health belief category was calculated to get the composite score. A composite score of ≥ 4 was considered “positive”, while a score of ≤ 3 was considered “negative” to the engagement in health behaviors to prevent colorectal cancer. Paired sample *t* tests were conducted on the pre- and post-scores to evaluate change in health beliefs. Spearman’s correlation was performed on data from the post-survey to evaluate the relationship between the number of program activities that an individual participated in and health belief composite scores.

### Information Session Surveys

Surveys to evaluate participant’s actions in response to the information sessions were conducted at the beginning of the second, third, and fourth WebEx sessions. The question, “Did you view any of the previous WebEx sessions,” was asked in order to direct those who had participated in a previous WebEx to the follow-up questions about actions taken relevant to that session’s content. Survey questions for each session are provided with the Supplementary Materials.

### VCE Colorectal Cancer Screening Rate Change

Data on colorectal cancer screening rates was provided by the major state health insurance company, Anthem Blue Cross Blue Shield. Historic claims data for colonoscopy, sigmoidoscopy, fecal occult blood test (FOBT), and fecal immunoassay test were used to evaluate the screening rate of VCE employees insured by Blue Cross Blue Shield in June 2016 before the CCFZ campaign was implemented and in June 2017 after the campaign.

Data was also collected on CCFZ information session attendance and colorectal cancer screening pledge signatures.

## Results

### Health Beliefs Pre-survey

Two hundred and eighty-eight VCE employees (33%) responded to the pre-program colorectal cancer beliefs survey, and 115 (13%) responded to the post-program survey. The lower post-survey response rate was largely due to survey response solicitation being conducted electronically rather than in person in 2017.

The majority of respondents of the first survey indicated positive health beliefs about each of the five belief categories. The questions within each health belief construct showed good internal consistency. Over 20% of respondents had negative responses for the categories of *barriers to screening* and *severity of colorectal*. Post-survey responses showed similar results (Table 1). A closer analysis of responses to the individual questions related to *barriers to screening* showed that feeling that doctor visits to detect colorectal cancer would be unpleasant was a significant barrier. In addition, feeling uncomfortable talking about colorectal cancer and the cost of screening were also barriers for about a quarter of respondents. Relevant to the survey results is the finding that perceived barriers to colorectal cancer screening has the greatest influence on screening behavior. Responses to questions related to *severity of colorectal cancer* indicated that although the majority felt a diagnosis of CRC would bring long lasting problems that would change their life, only slightly more than half of respondents (57.5%) indicated a fear of getting colorectal cancer. Additionally, a large majority (86%) felt that they would live longer than 5 years if diagnosed with the disease.

**Table 1:** Composite scores of health beliefs related to colorectal cancer

Health belief category	Pre-survey results (*N* = 288)			Post-survey results (*N* = 115)		
	Cronbach’s α	% positive^a^	% negative^b^	Cronbach’s α	% positive^a^	% negative^b^
Self-Efficacy	0.710	90%	10%	0.749	90%	10%
Benefit of screening	0.816	92%	8%	0.742	96%	4%
Barriers to screening	0.817	78%	22%	0.805	78%	22%
Susceptibility to colorectal cancer	0.832	85%	15%	0.852	82%	18%
Severity of colorectal cancer	0.715	74%	26%	0.686	76%	24%

^a^percent of responses with composite score ≥ 4

^b^percent of responses with composite score ≤ 3

Based on the findings of the pre-program survey, the communication plan incorporated messages from the NCCRT communications guidebook into planned Facebook and Twitter posts that were designed to alleviate these specific barriers to screening and provide accurate prognosis information.

Examples:

Although colon cancer is the second-leading cause of cancer death, it is also one of the most preventable with early detection.

There are several screening options available, including simple take-home options. Talk to your doctor about getting screened today!

### CCFZ Information Session Outcomes

One hundred and twenty-eight Extension employees, representing 81 unique individuals, participated in the four web-based information sessions conducted in 2016 (Average of 33 participants per session; range 20–37). Attendees represented 42 of the 107 counties in Virginia, with representation from each of the four Extension Districts. Sixty-two participants in session 2, 3, and 4 indicated that they had attended a previous session. Results from those who had attended a previous session showed that 50% (*n* = 31) of these respondents signed the colorectal cancer pledge, 42% (*n* = 26) took action to get screened, and 65% (*n* = 40) talked to someone about colorectal cancer. Over three-quarters of respondents had made changes in their diet to reduce their colorectal cancer risk. The most common dietary change was to increase fruit and vegetable consumption, followed by decreasing red meat intake, and increasing fiber intake. Forty percent of respondents also increased their physical activity. Specific changes in physical activity included beginning to exercise, increased exercise time or intensity of their exercise, and adding strength or flexibility training (Table 2).

**Table 2:** Diet and physical activity changes by information session participants

Dietary changes (session 3, *N* = 42)						
Increased fruit and vegetables	Increased fiber	Increased whole grains	Decreased red meat	Decreased processed meats	Decreased alcohol	Increased low-fat dairy/soy milk
76% (32)	48% (20)	31% (13)	50% (21)	43% (18)	12% (5)	21% (9)
Physical activity changes (session 4, *N* = 18)						
Began exercising	Increased exercise time or intensity	Added strength training	Added flexibility training			
22.2% (4)	50.0% (9)	22.2% (4)	33.3% (6)			

### Health Beliefs Post-survey Outcomes

One hundred and fifteen VCE personnel responded to the post-program health beliefs follow-up survey. Of these, forty had answered the pre-program survey. The vast majority (90%) of respondents were aware that VCE had joined the NCCRT 80% by 2018 initiative. Questions regarding their engagement with the CCFZ program activities indicated that 39% had participated in an information session, 38% viewed the webpage, 36% talked to a colleague, 46% saw the campaign poster, and 50% had signed the colorectal cancer pledge. Only 5% had seen a tweet, and 11% participated in no program activities. Twenty-one participants indicated that they had been screened in the past year. Of these, 12 people responded to the question of whether they got screened due to the 80% by 2018 initiative, 5 (42%) indicated that they had.

The analysis of mean change in health belief category composite scores among those who completed both the pre-and post-health beliefs survey showed that there was a significant increase in self-efficacy (4.01 ± 0.79 vs 3.74 ± 0.78, *ρ* = 0.029, *N* = 40). This increase strengthened when only those who indicated they had engaged in some way in the activities of the initiative were included in the analysis (*N* = 37; 4.15 ± 0.64 vs 3.81 ± 0.76, *ρ* = 0.006). There was no significant change in the other categories.

The number of CCFZ program activities that an individual participated in (0–6) was directly correlated with the HBM categories of self-efficacy, perceived benefits of screening, and inversely correlated with perceived barriers to screening. Higher scores for the latter category indicate a negative impact on engagement in healthy behaviors related to CRC prevention (Table 3).

**Table 3:** Correlation of initiative participation and health beliefs

Category	Self-efficacy	Perceived benefits	Barriers to screening
Correlation coefficient*	0.189	0.236	− 0.233
Sig (*ρ*)	0.05	0.012	0.013

*Spearman’s rho

### Change in VCE Screening Rate

The screening rate of VCE employees increased from 52.7 on June 2016 to 73.3% on June 2017.

## Discussion

The reduction of colorectal cancer incidence and mortality is a national priority [8]. Evidence-based, multicomponent strategies to increase colorectal cancer awareness and screening have been shown to be effective and are encouraged by the Centers for Disease Prevention and Promotion [18, 19]. With over 126 million US citizens in full-time employment at the end of 2017 [20], worksites are a ready vehicle for reaching a large portion of the population with key health messages and creating environments that promote healthy lifestyles. There is strong evidence that worksite wellness programs founded on evidence-based principles can impact employee lifestyle behaviors and health outcomes, and examples of their application to promote cancer screening exist [15, 21].

We demonstrated the successful implementation of a worksite colorectal cancer awareness program, the Colon Cancer–Free Zone (CCFZ), with Virginia Cooperative Extension (VCE). The program resulted in a 20% increase in employee colorectal cancer screening rates and changes in dietary and physical activity behaviors that are shown to reduce colorectal cancer risk. The CCFZ is a multicomponent program that combines various strategies to increase knowledge, dispel misinformation, improve self-efficacy, and remove barriers to action. The key strategies of the program include accessible information sessions provided by recognized experts, relevant repeated messages transmitted through multiple media platforms, a call to action via a colorectal cancer pledge, peer champions and testimonials, and visible administration support.

Due to the nature and size of the state Cooperative Extension service, most of the program activities were conducted virtually. The initial announcement of the CCFZ program was done by the administration during the annual VCE in person conference, and employees in county Extension offices were encouraged by the peer champions to attend the virtual information sessions together. Although the majority of individuals viewed the information sessions alone, fourteen county Extension offices attended one or more of the sessions in a group. The participation rate in the web-based sessions was approximately 9.3% (81 unique individuals out of 827 total Extension employees). This does not capture the total number of employees who were impacted by the CCFZ program in some way, however. Ninety percent of the 115 employees that responded to the post-program survey indicated they were aware of the CCFZ campaign, and over a third had seen information on the webpage, talked to a colleague about colorectal cancer, or seen the information poster. Half had responded to the call to sign the CCFZ pledge. Of all of the program strategies, Twitter appeared to be the least effective, with only 5% of survey respondents indicating they had seen a tweet. Of significance, the number of program strategies that a person was exposed to was directly associated with greater self-efficacy, a higher perception of the benefits of screening, and decreased barriers to screening.

Valuable information was gathered from the peer champions about their role in the program during informal interviews and a post-program survey. All of the champions were satisfied with the frequency and clarity of information they received from the program organizers and transmitted this information to colleagues in their district through email and announcements during district meetings. They confirmed that using multiple media platforms to transmit messages was necessary to reach more people and acknowledged that Twitter was the least effective as it is not commonly used by themselves or their colleagues. Key recommendations from the champions to increase program effectiveness were to have more peer champions, ensure support of program activities by administrators in the local Extension offices, and to encourage friendly competition between Extension locations by publicizing program participation by office location.

Subsequent to the completion of the Colon Cancer–Free Zone within VCE, the program was adapted for use by Cooperative Extension Agents at worksites in their service communities. The timeframe of the program was shortened to a two- to four-week period, and information sessions were condensed to two sessions. Key elements of the program were retained, however, including the campaign atmosphere of the program, recommendation of demonstrated buy-in by worksite administration, a relevant communication plan, peer support, participation incentives, and a call to action. A toolkit with implementation guides, templates, and communication guides has been developed and was introduced to Cooperative Extension agents. Agent program uptake, implementation, and outcomes are being evaluated.

## Electronic Supplementary Material

ESM 1 (DOCX 16 kb)

ESM 2 (DOCX 178 kb)

## References

[1] U.S. Cancer Statistics Working Group (2017). United States Cancer statistics: 1999–2014 incidence and mortality web-based report.

[2] World Cancer Research Fund International/American Institute for Cancer Research (2017). Continuous update project report: diet, nutrition, physical activity and colorectal cancer.

[3] Surveillance, epidemiology, and end results program (n.d.). Retrieved February 16, 2016, from http://seer.cancer.gov/statfacts/html/colorect.html

[4] Rawl SM, Menon U, Burness A, Breslau ES (2012). Interventions to promote colorectal cancer screening: an integrative review. Nurs Outlook.

[5] U.S. Department of Agriculture. National Institute of Food and Agriculture. Cooperative extension history. Retrieved January 27, 2018, from https://nifa.usda.gov/cooperative-extension-history

[6] U.S. Cooperative Extension Service (2014) Cooperative Extensions’ National Framework for Health and Wellness. Retrieved May 7, 2017, From http://www.aplu.org/members/commissions/food-environment-and-renewable-resources/CFERR_Library/national-framework-for-health-and-wellness/file?id=5134

[7] U.S. Health and Human services (2014) National prevention strategy fact sheet. Retrieved 2017, From https://www.surgeongeneral.gov/priorities/prevention/strategy/national-prevention-strategy-fact-sheet.pdf

[8] National Colorectal Cancer Roundtable. http://nccrt.org/what-we-do/80-percent-by-2018/. Accessed on January 3, 2019

[9] Crookes DM, Njoku O, Rodriguez MC, Mendez EI, Jandorf L (2014). Promoting colorectal cáncer screening through group education in community-based settings. J Cancer Educ.

[10] Greenwald B (2006). Promoting community awareness of the need for colorectal cancer screening: a pilot study. Cancer Nurs.

[11] Bagai A, Parsons K, Malone B, Fantino J, Paszat L, Rabeneck L (2007). Workplace colorectal cancer-screening awareness programs: an adjunct to primary care practice. J Community Health.

[12] Hannon PA, Vu T, Ogdon S, Fleury EM, Yette E, Wittenberg R, Celedonia M, Bowen DJ (2013). *I*mplementation and process evaluation of a workplace colorectal cancer screening program in eastern Washington. Health Promot Pract.

[13] Harris JR, Parrish AT, Kohn M, Hammerback K, McMillan B, Hannon PA (2015). Promoting employee health through an American Cancer Society program, the CEPs Challenge, Washington State, 2013–2015. Prev Chronic Dis.

[14] Goetzel RZ, Shechter D, Ozminkowski RJ, Marmet PF, Tabrizi MJ, Roemer EC (2007). Promising practices in employer health and productivity management efforts: findings from a benchmarking study. J Occup Environ Med.

[15] O’Donnell M, Bishop C, Kaplan K (1997). Benchmarking best practices in workplace health promotion. Am J Health Promot.

[16] Hughes AG, Watanabe-Gallowy S, Schnell P, Soliman AS (2015). Rural-urban differences in colorectal cancer screening barriers in Nebraska. J Community Health.

[17] American Cancer Society. 80% by 2018 recommended messaging to reach the unscreened: 2016 communications guidebook

[18] Tangka FKL, Subramanian S, Hoover S, Royalty J, Joseph K, DeGroff A, Joseph D, Chattopadhyay S (2017). Costs of promoting cancer screening: evidence from CDC’s colorectal cancer control program (CRCCP). Eval Program Plann.

[19] Cancer screening: multicomponent interventions – colorectal cancer. (2016, August) The community guide. https://www.thecommunityguide.org/findings/cancer-screening-multicomponent-interventions-colorectal-cancer

[20] United States Department of Labor Bureau of Labor Statistics. Retrieved July 20, 2018 from https://data.bls.gov/cgi-bin/surveymost

[21] Kent K, Gotzel RZ, Roemer EC, Prasad A, Freundlich N (2016). Promoting healthy workplaces by building cultures of health and applying strategic communications. JOEM.

