# Supplementary material for: Creating a Workplace Culture of Preventive Health: Process and Outcomes of the Colon Cancer–Free Zone at Virginia Cooperative Extension
Source: J Cancer Educ. 2019 Jul 15;35(6):1135–40. doi: 10.1007/s13187-019-01569-4 (PMC7679323; doi:10.1007/s13187-019-01569-4)
Supplement: Supplementary file 1 — (DOCX 16 kb) [file 13187_2019_1569_MOESM1_ESM.docx]

| CCFZ Information Session Survey Questions^a^ |
| --- |
| *Questions asked at the beginning of sessions 2, 3, & 4*  Since viewing the previous WebEx have you:   - signed the Colorectal Cancer Free Zone Pledge? - taken any action toward getting screened? - talked to anyone else about colorectal cancer? |
| *Questions asked at the beginning of session 3*  Since participating in the previous WebEx, what lifestyle changes have you made to decrease your risk for colorectal cancer?   - Increased fruit and vegetable consumption. - Increased fiber intake. - Increased whole grain consumption. - Decreased red meat intake. - Decreased consumption of processed meats. - Decreased alcohol intake. - Increasing low-fat dairy or calcium fortified soy milk. - Increased physical activity. - Other. |
| *Questions asked at the beginning of session 4*  Since participating in the previous WebEx, what physical activity lifestyle changes have you made to decrease your risk for colorectal cancer?   - Have begun exercising. - Have increased the time or intensity of exercise. - Have added strength training to exercise routine. - Have added flexibility training to exercise routine. - Other |
| ^a^questions were asked about actions taken in response to the previous information session attended |
