# Supplementary material for: Creating a Workplace Culture of Preventive Health: Process and Outcomes of the Colon Cancer–Free Zone at Virginia Cooperative Extension
Source: J Cancer Educ. 2019 Jul 15;35(6):1135–40. doi: 10.1007/s13187-019-01569-4 (PMC7679323; doi:10.1007/s13187-019-01569-4)
Supplement: Supplementary file 2 — (DOCX 178 kb) [file 13187_2019_1569_MOESM2_ESM.docx]

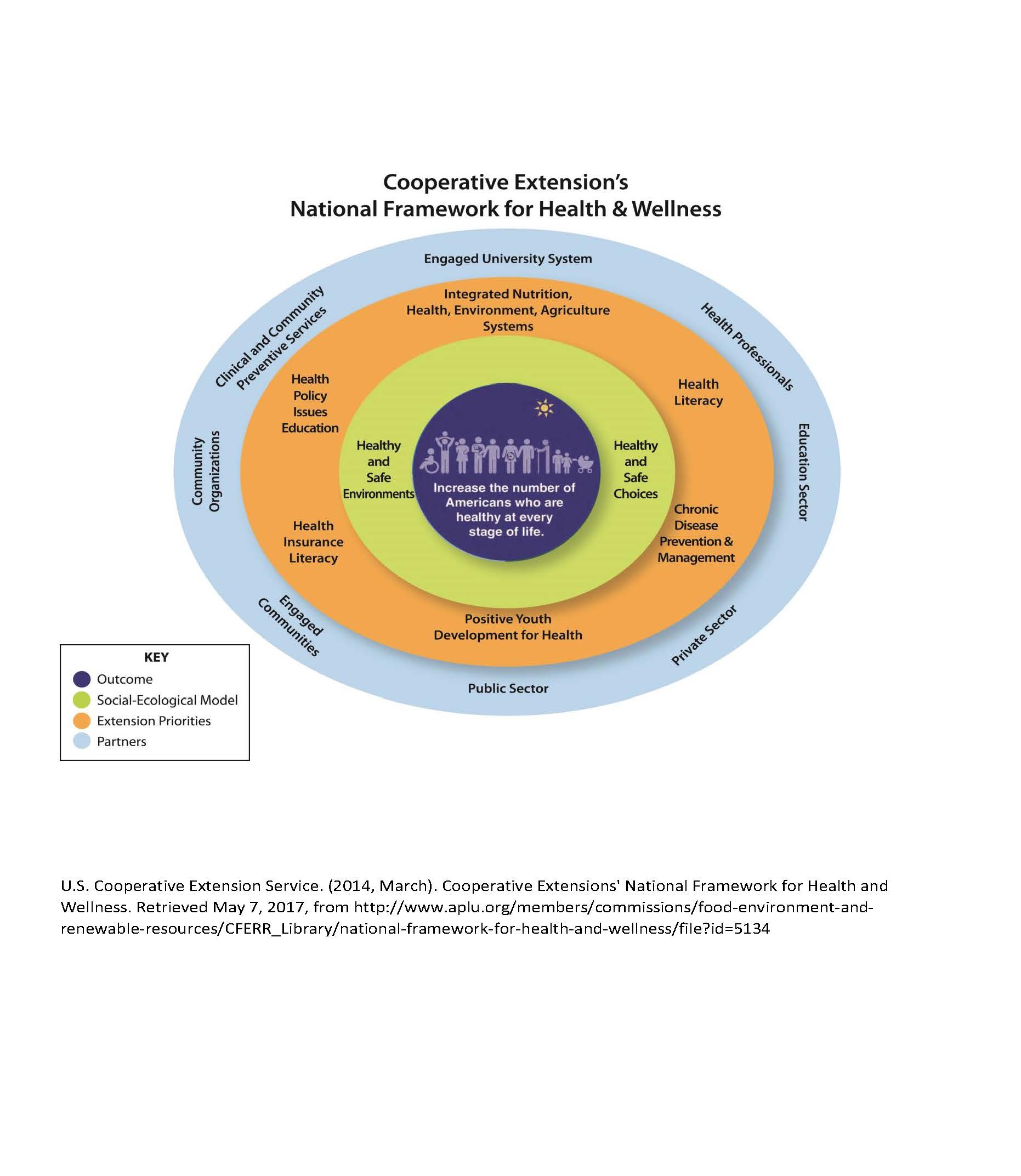


From: U.S. Cooperative Extension Service. (2014, March). Cooperative Extensions' National Framework for Health and Wellness. Retrieved February 9, 2019, from <http://www.aplu.org/members/commissions/>

food-environment-and-renewable-resources/CFERR_Library/national-framework-for-health-and-wellness/file?id=5134
